# Development and validation of a questionnaire for measuring team cohesion: the Erlangen Team Cohesion at Work Scale (ETC)

**DOI:** 10.1186/s40359-024-01583-2

**Published:** 2024-02-22

**Authors:** Marietta Lieb, Yesim Erim, Eva Morawa

**Affiliations:** Department of Psychosomatic Medicine and Psychotherapy, Friedrich-Alexander-University Erlangen-Nürnberg (FAU), Schwabachanlage 6, 91054 Erlangen, Germany

**Keywords:** Validation, Questionnaire, Team cohesion, Work

## Abstract

**Background:**

Team cohesion is a crucial factor when it comes to job satisfaction and turnovers. However, in Germany, economic measures for team cohesion are scarce. The aim of this study was to develop and validate an economic self-report questionnaire for measuring team cohesion in a work setting in health care.

**Methods:**

The questionnaire was developed in a stepwise procedure. After item analysis, exploratory factor analysis was conducted to assess factor structure. Reliability was tested via internal consistency. To assess convergent and divergent validity, we applied the Copenhagen Psychosocial Questionnaire (COPSOQ), the Perceived Cohesion Scale (PCS), the ENRICHD Social Support Inventory (ESSI), the Effort-Reward Imbalance Scale (ERI) and the Patient Health Questionnaire (PHQ-4), respectively.

**Results:**

The pilot version was tested in a sample of *n* = 126 adult nurses. Item analysis resulted in a total of 13 items for the final version. Exploratory factor analysis indicated a two-factor structure. Internal consistency for the two subscales was good, with α = 0.88 and α = 0.84, respectively. Convergent validity with the subscales of COPSOQ and PCS was moderate to high (*r* =.26– *r* =.64). For divergent validity, correlations with the ESSI were low (*r* =.01– *r* = -.09). We further found significant correlations with depression symptoms (r=-.22– *r*=-.37), as well as reward (ERI) (*r* =.41 -*r* =.47) and effort (ERI) (*r*=-.20 - r = -.24).

**Conclusions:**

We developed and validated the Erlangen Team Cohesion at Work Scale (ETC), a self-report measure for team cohesion with very good psychometric properties. Due to its economic deployment, it is suitable for measuring team cohesion in work settings, especially in health care.

**Supplementary Information:**

The online version contains supplementary material available at 10.1186/s40359-024-01583-2.

## Background

During the COVID-19 pandemic, working conditions have deteriorated massively for health care workers, due to higher workload, uncertainty about the disease, high number of critical cases, risk of infection and many more [1]. Especially for nurses, this development has led to a dramatic increase in turnover rates, an escalation of an already existing shortage [2, 3]. Turnovers again impact the remaining staff, starting a vicious circle of increasing burden and lack of staff. Therefore, it has become of upmost importance to identify factors buffering this trend. One factor of importance found in the literature is team cohesion and social connectedness within the working team. In a study on emergency nurses, low social support and low connectedness to the team were identified as factors contributing to turnover intention [4]. Team cohesion was further found to be an influencing factor for both job satisfaction [5] and the intention to remain employed [6].

Team cohesion has also been examined in health care workers in general and was found to be a crucial factor for work performance and satisfaction. Öhman et al. [7] found team cohesion to be a substantial component for work climate and overall work satisfaction for health care workers in elderly care. For community mental health teams, higher team cohesion lead to higher effectiveness [8].

Team cohesion is a highly universal construct and does not solely apply to health care workers, but also to other working and economic settings, to sports and military contexts as well as group therapy [9–12]. Beneficial effects of team cohesion were also found there. For instance, group cohesion was related to lower job stress and higher job satisfaction in US company employees [13], while in sports teams and military units higher connectedness and cohesion was found to be associated with less depression and less anxiety symptoms [14, 15].

Due to its universality, team cohesion has a variety of definitions [12]. For instance Forsyth [16] regarded cohesion as a “glue” or interpersonal forces that bind group members together. He describes group cohesion as the integrity, solidarity, social integration and unity of a group, where social exchange is an important instrument to keep the group intact and move towards a goal [16]. In another common definition, team cohesion is described as a “dynamic process that is reflected in the tendency for a group to stick together and remain united in the pursuit of its instrumental objectives and/or for the satisfaction of member affective needs” [11, 17]. According to both definitions, cohesion implies interpersonal relationships with a strong sense of unity within the group as well as cooperation and goal commitment, two factors that are commonly called “social cohesion” and “task cohesion” [10–12, 17, 18]. Other researchers also describe other aspects inherent to team cohesion, such as “attraction to the group”, “belongingness”, “group pride”, “shared identity” and “morale” [9, 10, 12], leading to a diversification of the construct.

By now, a variety of international measurement instruments with different operationalizations of team cohesion have been developed, some of them applicable to work [19, 20], sports [21, 22], group therapy [23, 24] or other [25, 26] settings. However, German questionnaires measuring team cohesion in a workplace setting are rare. Those that do exist are mostly overly comprehensive and time-consuming e.g. the TKI (Team Climate Inventory) [27], the FITOR (Fragebogen zur individuellen, Team und organisationellen Resilienz) [28], the COPSOQ (Copenhagen Psychosocial Questionnaire) [29] and the FAT (Fragebogen zur Arbeit im Team) [30]. Especially in health care settings, where workload is high and time is short, brief and economic measures of team cohesion are essential. Therefore, we aimed to develop and validate an economic self-report questionnaire for measuring team cohesion in a work and health care setting in Germany. For the construction of the questionnaire, we aimed to focus on already existing definitions and questionnaires described above. We further aimed to assess its factor structure by using exploratory factor analysis and to evaluate its psychometric properties such as reliability and convergent and divergent validity.

## Methods

### Setting and data collection

The data originated from the project “Stress-Monitor 2”, a project developed and conducted at the Department of Psychosomatic Medicine and Psychotherapy at the University Hospital in Erlangen. The primary aim of this study was to assess the association between team cohesion, working ability and biological stress markers in adult nurses. As a sub-study, we aimed to develop a new questionnaire for team cohesion. We recruited nurses at the University Hospital of Erlangen and the “Malteser Waldkrankenhaus” in Erlangen via flyers, intranet advertisements and personal recruitment. Participants had to fill-out an online questionnaire that could be accessed via Online-Link/QR-Code. All participants provided their online informed consent prior to the survey (The participants had to tick a box to consent to the anonymous collection, analysis and publication of data). Participants could either terminate study participation after completing the online survey (part 1) or proceed with an additional study measuring salivary cortisol (part 2), receiving an incentive of 20€ (results of part 2 are displayed elsewhere). In order to merge the results from the two study parts, participants had to create a code (day of birthday, first letter of birth place etc.) in the online survey. However, the identity of the participants remained completely anonymous during part 1, since identification was only possible in combination with the participation in part 2. No feedback on personal results was thus possible when participating only in part 1. After the online survey, the contact details of the leading psychological investigator were provided. In case psychological support was needed, the participants were able to contact us for a referral to the psychosomatic outpatient clinic. Recruitment took place between November 2022 and June 2023. The study was reviewed and approved by the local ethics committee of the Medical Faculty of the Friedrich-Alexander-University Erlangen-Nürnberg according to the Declaration of Helsinki.

### Procedure and analysis

The construction of the questionnaire and the evaluation of its psychometric properties was performed in a stepwise procedure. A quick overview of the steps can be viewed in Table 1.

**Table 1:** Stepwise procedure for the development of the Erlangen Team Cohesion at Work Scale

Step 1	- Comprehensive review of the literature - Generation of an initial item pool
Step 2	- Expert rating according to comprehensibility, relevance and adequacy - Generation of a pilot version
Step 3	- Testing the pilot version in a sample of adult nurses
Step 4	- Statistical testing: Item analysis, exploration of factors structure and reduction of item count, testing of psychometric properties (reliability, validity) - Generation of final version

Step 1 included a comprehensive literature research on the term “cohesion” (e.g. team cohesion, social cohesion, group cohesion, etc.) and already existing measurement instruments (cohesion questionnaire, measuring team/social/group cohesion etc.) in both English and German on PubMed and Google Scholar. The first item pool was generated on the basis of literature recommendations, definitions of team cohesion (e.g. Forsyth [16], Bollen & Hoyle [31] and Vanhove & Herian [11]) and already existing questionnaires (TKI, FITOR, COPSOQ, FAT etc [27–30])..

During step 2, a team of 10 experts, consisting of a total of 6 researchers/psychologists, 1 psycho-oncologist, 2 medical students and 1 professor for psychosomatics rated the item pool according to comprehensibility, relevance and adequacy on a scale ranging from 1 (excellent) to 6 (insufficient). The items were ranked according to their average score, followed by an item selection and reduction of the item pool. Suggested word alterations of single items and further adaptations were discussed in a group of 2 expert psychologists and adapted if considered relevant. In a final phase, the pilot version of the questionnaire was generated.

During step 3, the resulting pilot version was tested in a sample of *n* = 126 nurses. The questionnaire could be accessed via online link and was available for the duration of 7 months.

During step 4 we conducted item analysis (assessment of missings, item difficulty, ceiling and floor effects and item discrimination) and used statistical tests on factor structure to reduce item count (exploratory factor analysis). We evaluated the psychometric properties of the questionnaire by assessing reliability and convergent and divergent validity and terminated with a final, modified version of the questionnaire (for more details please see Data Analysis). The final validated German version was then translated to English according to the guidelines for cross-cultural adaptation of self-report measures (including forward translation by two native speakers, synthesis, back translation, and discussion by experts) [32].

### Measures

Besides the pilot version of our questionnaire, we applied 10-items of the Copenhagen Psychosocial Questionnaire (COPSOQ) [29] assessing relations to colleagues and superiors. These 10 items included the subscales “support at work” (4 Items), “Feedback” (2 Items), “Quantity of Social Relations” (1 Item), “Sense of community” (2 Items) and “Unfair treatment” (1 Item). The original COPSOQ is an 84 item self-report questionnaire to measure psychosocial factors at work. For most of the 84 items and 31 scales, reliability and validity is good to very good [29]. For the chosen subscales we found the following Cronbach alpha’s in our sample: “Support at work”: α = 0.79, “Feedback”: α = 0.66 and “sense of community”: α = 0.81. The COPSOQ was developed by the work group of Nübling et al. [33] and is available in several languages by now.

We further applied the German version of the Perceived Cohesion Scale (PCS) that was originally developed by Bollen & Hoyle [31, 34]. The PCS is an economic 6-item measure on perceived cohesion, which was translated to German and validated by our research group [35]. We found very good reliability (α = 0.93-0.94) and validity.

We also employed the German Version of the ENRICHD Social Support Inventory (ESSI) by Kendel et al. [36], a 5-item measure to assess perceived emotional and social support. The questionnaire shows good psychometric properties with a reported internal consistency of α = 0.89 (for our sample we found α = 0.90) and satisfying construct validity.

In addition, we used the ultra-short version of the Patient Health Questionnaire (PHQ-4 [37]), a screener for depression and anxiety. The PHQ-4 consists of 4 items, while the first two of them form the PHQ-2 [38] assessing depression symptoms, while the last two form the GAD-2 [39], measuring anxiety symptoms. The sum score for the PHQ-4 ranges from 0 to 12 and has a cut-off at ≥ 6, while the PHQ-2 and the GAD-2 each have a cut-off at ≥ 3, indicating clinically relevant depression and anxiety symptoms, respectively. The internal consistencies for the scales are reported as follows: α = 0.78 (PHQ-4), α = 0.75 (PHQ-2), α = 0.82 (GAD-2) [37]. For our sample we found equal values: α = 0.81 (PHQ-4), α = 0.76 (PHQ-2), α = 0.74 (GAD-2).

The German Version of the Effort-Reward Imbalance Scale (ERI) [40], a 10-item questionnaire, was employed to assess effort (E) and reward (R) as well as the degree of imbalance between them (Effort-reward ratio = ERR = E/R*C; C = 3/7 = 0.4286). A ratio of > 1 means that perceived effort exceeds perceived rewards (= effort-reward-imbalance). Internal consistencies were acceptable for both scales with a reported Cronbach’s Alpha of α = 0.74 for effort and α = 0.79 for reward. Both discriminant validity and criterion validity is established. In our sample the Cronbach Alpha’s were as follows: α = 0.67 (effort) and α = 0.67 (reward).

We also assessed sociodemographic data on age, sex, marital status (single, married, in relationship, separated, divorced, widowed), children (yes/no), migration background, working experience (< 3 years, 3–6 years, > 6 years, no experience in patient care) and working hours (full-time/ part-time).

### Data analysis

Statistical Analyses were performed with SPSS for Windows, Version 28. For descriptive statistics we depicted frequencies, mean values, standard deviations and ranges. For correlations we used the Pearson correlation coefficient. For item analysis we assessed the amount of missings, item difficulty (0.2 < x > 0.8, [41, 42]), ceiling and floor effects and item discrimination (< 0.3 [41]). Kaiser Meyer Olkin (KMO) statistics for sampling adequacy, the Bartlett’s test of sphericity as well as the Anti Image correlations were used to test for the appropriateness of factor analysis. We then conducted an exploratory factor analysis via principal component analysis with varimax rotation. We used the Kaiser criterion for eigenvalues > 1 in order to decide about the factor structure. We conducted a reliability analysis in order to analyze Cronbach’s Alpha for internal consistency. We further conducted several tests to determine validity: Convergent validity was determined by correlating our questionnaire with the subscales of the German COPSOQ [29] and the PCS [31, 34] via Pearson correlation. The higher the correlation, the higher the convergent validity. Equally, divergent validity was determined via Pearson correlation with the ESSI [36], the PHQ-4 [37] and the ERI [40]. Test assumptions were assessed in advance and statistical tests were selected accordingly. A significance level of *p* <.05 was predefined for all analyses.

We aimed at a minimum sample size of *n* = 100. Considering a power of 0.8 and a significance level of *p* <.05, a sample size of 85 is required to reveal a significant correlation of *r* =.30. For test analysis, a minimum sample size of *n* = 100 is required [43, 44]. In addition, a sample of 100 is considered sufficient for exploratory factor analysis, when the communalities of all items are h²>0.50 [45].

## Results

### Results of step 1–2: generation of itempool, expert rating and pilot version

Following the literature research, the first item pool consisted of a total of *n* = 51 items to be rated on a 6-point scale (1 = very good to 6 = very bad) according to comprehensibility, relevance and adequacy by 10 experts. The total averaged rating scores of the items ranged between 1.03 and 2.04. As recommended by the majority of the expert team, we first discarded all items concerning the relationship with the superior in order to increase homogeneity of the questionnaire. In addition, we dismissed all duplicates/items with similar wording. We further discussed and adapted suggested word alterations in a team of two expert psychologists. The final item selection and reduction was conducted according to item rank (app. 30% of the initial item pool) which resulted in a pilot version of *n* = 15 items. The experts agreed on a response format of a 5 Point-Likert scale (0 = totally disagree to 4 = totally agree). The pilot version can be viewed in the additional file 1.

### Results of step 3: testing the pilot version in a sample of adult nurses

The pilot version was tested in a sample of *n* = 126 adult nurses. More than three quarters of the sample was female (79.4%), the mean age was M = 39.33 (SD = 12.86). Most participants were either married (34.9%), single (27.8%) or in a relationship (27.0%). Almost two thirds (61.9%) of the participants had no children. For more details of the sample characteristics, please view Table 2.

**Table 2:** Sample characteristics

		*N* = 126
**Gender**	Female	100 (79.4%)
	Male	26 (20.6%)
**Age**	M (SD), range	39.33 (12.86), 18–66
**Age Groups**	18–30	39 (31.0%)
	31–40	32 (25.4%)
	41–50	22 (17.5%)
	51–66	33 (26.25)
**Marital Status**	Single	35 (27.8%)
	Married	44 (34.9%)
	In a relationship	34 (27.0%)
	Separated	2 (1.6%)
	Divorced	8 (6.3%)
	Widowed	3 (2.4%)
**Children**	Yes	48 (38.1%)
	No	78 (61.9%)
**Migration background**†	Yes	17 (13.5%)
	No	109 (86.5%)
**Working experience**	< 3 years	13 (10.3%)
	3–6 years	16 (12.7%)
	> 6 years	95 (75.4%)
	Missing	2 (1.6%)
**Working hours**	Full-time	66 (52.4%)
	Part-time	60 (47.6%)

Note: †The participant or at least one parent did not have german citizenship by birth; M = mean, SD = standard deviation

### Results of step 4: statistical testing and final version

#### Item analysis

For item analysis, we first recoded inverse-coded items. We did not have to dismiss any items due to missings, since no item had missing values > 0.8%: 10 items had no missings at all, while 5 items had only one missing in total each (= 0.8%). We had to discard 2 items (13.33%) due to inadequate item difficulty (0.2 < x > 0.8, [41, 42]). No items had to be excluded due to ceiling and floor effects, since those in question had already been identified and discarded in the previous step. No item had to be discarded due to low item discrimination (< 0.3 [41]).

#### Exploratory Factor Analysis

After statistical item analysis and selection, we conducted an exploratory factor analysis with the remaining *n* = 13 items (see additional file 1). According to the KMO statistic (0.91), the Bartlett test of sphericity (*p* <.001) as well as the Anti Image correlations (> 0.5), the requirements for principal component analysis were met [46]. The sample size of *n* = 126 was also sufficient to conduct factor analysis, since all communalities had values h²>0.50 [45]. Factor analysis extracted two factors with eigenvalues > 1, suggesting a two-factor solution. The factor loadings of the rotated component matrix as well as all item scores are displayed in Table 3. The final version can be found in the additional file 2.

**Table 3:** Factor loadings and item scores of the final version of the Erlangen Team Cohesion at Work Scale

Items			Itemscores		
	**Factor loadings**		**M (SD)**	**Item difficulty**	**Item discrimination**
	**Factor 1**	**Factor 2**			
Factor 1: Collegial Solidarity (CS)			2.77 (0.67)		
We support each other	**0.60**	0.51	3.09 (0.78)	0.77	0.73
We treat each other with respect	**0.71**	0.41	2.86 (0.84)	0.71	0.74
We can rely on each other	**0.77**	0.31	2.90 (0.88)	0.72	0.71
There is a fair distribution of workload within the team	**0.71**	< 0.01	2.29 (0.91)	0.57	0.46
There is a good communication within the team	**0.78**	0.19	2.60 (0.84)	0.65	0.64
We stick together	**0.78**	0.31	2.88 (0.85)	0.72	0.72
Factor 2: Unity and problem management (UPM)			2.71 (0.68)		
There are members of the team that are being excluded	0.30	**0.65**	2.66 (1.04) †	0.66	0.59
Everyone is left to work on their own	0.04	**0.50**	2.86 (0.94) †	0.72	0.32
Everyone is free to express their opinion openly	0.30	**0.68**	2.90 (1.01)	0.72	0.61
We handle problems in a constructive manner	0.49	**0.60**	2.52 (0.90)	0.63	0.70
There is a sense of “we” among us	0.53	**0.63**	2.64 (1.02)	0.66	0.75
In case of disagreements, we usually find a good compromise	0.51	**0.63**	2.55 (0.83)	0.63	0.75
New team members are quickly integrated into the team	0.08	**0.74**	2.87 (0.90)	0.71	0.49
Total			2.74 (0.63)		

Note: †Mean after recoding; the main factor loadings are depicted in bold

#### Internal consistency

The scale “Collegial Solidarity” (CS) revealed a Cronbach’s Alpha of α = 0.88 while the scale “Unity and Problem Management” (UPM) had a Cronbach’s Alpha of α = 0.84, suggesting high internal consistency for both scales. Cronbach’s Alpha for the total scale was α = 0.91. There was a high correlation between the two factors (*r* =.75).

#### Convergent validity

Convergent validity was determined by correlating the two subscales as well as the total sum score of the Erlangen Team cohesion at Work Scale with two existing instruments for team cohesion, the COPSOQ and the PCS. The correlations coefficients are depicted in Table 4.

**Table 4:** Pearson Correlations between the Erlangen Team Cohesion at Work Scale and the subscales of the COPSOQ and the PCS.

	CS	UPM	Total sum score
COPSOQ; Support at work	0.51**	0.63**	0.61**
COPSOQ: Feedback	0.31**	0.36**	0.36**
COPSOQ: Quantity of Social relations	0.21*	0.38**	0.34**
COPSOQ: Sense of community	0.45**	0.60**	0.56**
COPSOQ: Unfair treatment	− 0.39**	− 0.46**	− 0.45**
PCS: Sense of belonging	0.46**	0.61**	0.56**
PCS: Morale	0.51**	0.64**	0.62**

Note: *p <.05, **p <.001; CS = Collegial Solidarity; UPM = Unity and problem management

#### Divergent validity

For assessing divergent validity, we conducted correlations of the two subscales with the sum score of ESSI, PHQ-4 and ERI. Results are displayed in Table 5.

**Table 5:** Pearson correlations between the Team Cohesion at Work Scale and the ESSI, PHQ-4 and ERI

	CS	UPM	Total sum score
ESSI	− 0.087, *p* =.332	0.013, *p* =.890	− 0.051, *p* =.573
PHQ-4: Sum score	− 0.162, *p* =.070	− 0.330, *p* <.001	− 0.266, *p* =.003
PHQ-2: Depression	− 0.220, *p* =.013	− 0.366, *p* <.001	− 0.321, *p* <.001
GAD-2: Anxiety	− 0.073, *p* =.416	− 0.227, *p* =.012	− 0.158, *p* =.081
ERI: Effort	− 0.201, *p* =.024	− 0.224, *p* =.013	− 0.239, *p* =.008
ERI: Reward	0.460, *p* <.001	0.408, *p* <.001	0.470, *p* <.001
ERI: Effort-reward-ratio	− 0.441, *p* <.001	− 0.422, *p* <.001	− 0.473, *p* <.001

Note: CS=Collegial Solidarity; UPM=Unity and Problem management

#### Descriptive characteristics of the Erlangen Eeam Cohesion at Work Scale

In Table 6, descriptive characteristics of the two factors of the Erlangen Team Cohesion at Work Scale are displayed according to gender, age, marital status, children (yes/no), migration background (yes/no), working experience and working hours.

**Table 6:** Descriptive characteristics of the Erlangen Team Cohesion at Work Scale

		CS (Mean, SD)	UPM (Mean, SD)
Gender	Female	2.75 (0.68)	2.68 (0.70)
	Male	2.81 (0.63)	2.85 (0.61)
Age Groups	18–30	2.81 (0.65)	2.81 (0.65)
	31–40	2.66 (0.61)	2.48 (0.76)
	41–50	2.70 (0.83)	2.70 (0.73)
	51–66	2.88 (0.65)	2.84 (0.56)
Marital Status	Single	2.81 (0.75)	2.82 (0.72)
	Married	2.79 (0.60)	2.68 (0.61)
	In a relationship	2.64 (0.66)	2.59 (0.75)
	Separated	3.42 (0.82)	3.50 (0.51)
	Divorced	2.65 (0.63)	2.55 (0.47)
	Widowed	3.44 (0.54)	3.29 (0.43)
Children	Yes	2.84 (0.62)	2.68 (0.66)
	No	2.73 (0.70)	2.74 (0.70)
Migration background†	Yes	2.43 (0.60)	2.24 (0.61)
	No	2.82 (0.67)	2.78 (0.67)
Working experience	< 3 Years	2.83 (0.64)	2.56 (0.84)
	3–6 Years	3.07 (0.63)	2.97 (0.60)
	> 6 Years	2.71 (0.68)	2.69 (0.68)
Working Hours	Full-time	2.72 (0.72)	2.61 (0.70)
	Part-time	2.82 (0.61)	2.84 (0.65)

Note: †The participant or at least one parent did not have German citizenship by birth; CS = Collegial Solidarity; UPM = Unity and Problem Management

## Discussion

In this study we developed and validated an economic self-report questionnaire for measuring team cohesion in a health care setting in Germany, the Erlangen Team Cohesion at Work Scale. This questionnaire measures two factors of team cohesion, Collegial Solidarity (CS) and Unity and Problem Management (UPM), both with very good internal consistencies (α = 0.88 and α = 0.84) as well as adequate convergent and divergent validity. Due to the written instruction and standardized calculation and interpretation of results, implementation and evaluation objectivity is ensured. A comprehensive literature research and feedback on content by psychology experts further indicate content validity. Our findings suggest that the Erlangen Team Cohesion at Work Scale is a reliable and valid instrument to measure team cohesion at work.

Equal to most former research [9, 10], we were able to identify more than one factor for team cohesion, underlining the multifacetedness of this construct. Although our two factors CS and UPM cannot be distinctly assigned to “social cohesion” and “task cohesion”, two aspects of cohesion suggested previously [9, 10], they do show similarities: The subscale CS comprises mutual support (“We support each other”), respectful treatment (“We treat each other with respect”), trust (“We can rely on each other”), communication (“There is a good communication within the team”), equality (“There is a fair distribution of workload within the team”) and unity (“We stick together“), all crucial aspects of maintaining interpersonal relationships within a team as it is the case for “social cohesion” [9]. While the subscale UPM also includes features of “social cohesion” such as a sense of “we” (“There is a sense of “we” among us”), it further comprises aspects of how teams interact in order to reconcile conflicts, such as constructive problem solving (“We handle problems in a constructive manner”), finding compromises (“In case of disagreements, we usually find a good compromise”), free expression of opinion (“Everyone is free to express their opinion openly”), integration (“New team members are quickly integrated into the team”), inclusion (“There are members of the team that are being excluded” = inversely coded) and collaborative working (“Everyone is left to work on their own” = inversely coded) and thus achieve goals in the long run, which remotely resembles “task cohesion” [9]. Literature suggests that “social and task cohesion” are very strongly interlinked and that social cohesion also might be an antecedent for task cohesion, which makes it difficult to distinguish these two aspects empirically [10, 11]. These conceptual overlaps are also visible in the high correlation between our two factors (r =.75), as well as in the nearly similar factors loadings for “There is a sense of “we” among us” (.53 vs..63), “In case of disagreements, we usually find a good compromise” (.51 vs..63), and “We handle problems in a constructive manner” (.49 vs..60).

Research also found other aspects when examining team cohesion: Forsyth [16] described cohesion as the integrity, solidarity, social integration and unity of group. Content-related aspects that also can be found in our questionnaire, both for CS (e.g. “We stick together” for solidarity) and UPM (“There are members of the team that are being excluded” and “Everyone is left to work on their own” for social integration and “There is a sense of “we” among us” for unity of group).

Bollen and Hoyle [31] restricted their conceptualization of team cohesion to the two dimensions “sense of belonging” and “feelings of morale”. Sense of belonging is defined as the cognitive dimension, fundamental to identification with the group and relationships between group members, while “feelings of morale” are seen as the affective component, implying emotional consequences of this belonging [31]. When testing our questionnaire for convergent validity, we found CS and UPM to correlate strongly with “sense of belonging” (CS: *r* =.46, UPM: *r* =.61) and “feelings of morale” (CS: *r* =.51, UPM: *r* =.64), depicting a similarity in content. We further observed moderate to high correlations with the COPSOQ measuring relationships with colleagues and supervisors. Highest correlations of CS and UPM were found with the COPSOQ scales “support at work” and “sense of community”, suggesting high similarities between the scales, while the high negative correlation with “unfair treatment” suggests high dissimilarity. As for “feedback” and “quantity of social relations” correlations might be lower due to lesser similarity in content: “Feedback”, for instance, includes items on how often feedback is received on one’s work from colleagues and supervisors, while “quantity of social relations” depicts the frequency of communication during work.

When testing for divergent validity, we found no association between the two factors of the team cohesion at work scale and the ESSI. Although both instruments measure social aspects, the Erlangen Team Cohesion at Work Scale measures cohesion in the working context, while the ESSI measures social support in the private environment, two distinct constructs, suggesting high divergent validity. When correlating the Erlangen Team Cohesion at Work Scale with the PHQ-4, we received mixed results. For depression symptoms we detected moderate correlations. As depicted above, lower depression scores were previously found to be related to cohesion in several contexts, in team sports [15], in military [14], but also in social and community environments in general [47–49]. In a work place setting, lower depression symptoms have been shown to be related with higher “social capital” [50, 51], a construct related to social cohesion, since it involves social networks, reciprocity, trust and cooperation [52]. The associations between depression symptoms and cohesion seems noteworthy, considering the increased occurrence of depression in nurses [53] and the rising prevalence of diagnoses in general in recent years [54]. Although no cause-and-effect relation is established yet, cohesion might have a mitigating role for depression symptoms and be a protective factor for mental health in general. Especially since depression symptoms are tightly connected with intention to leave the job in nurses [55] and in employees in general [56], cohesion should be measured and monitored regularly in order to intervene timely.

For anxiety, only a significant correlation was found for UPM. Anxiety has also previously been associated with cohesion in different settings: Higher cohesion was related to lower anxiety in a military setting [14], while community cohesion was found to be a protective mechanism against health anxiety during the first national COVID-19 lockdown [57]. Possibly in our study, the way of how teams/and or communities manage conflicts (= UPM) has a greater association with anxiety than respectful treatment (“We treat each other with respect”), trust, (“We can rely on each other”) and mutual support (“We support each other“) (= CS).

We further observed moderate to high correlations with the effort and reward scale of ERI: Higher CS and UPM were associated with lower effort, higher reward and lower effort-reward-ratio: Higher team cohesion might act as a buffer against physical and mental exertion, as it does for mental distress described above. Previous research [58] that found effort-reward imbalance to be significantly negatively associated with job satisfaction underlines this conclusion.

### Summary and limitations

With this study we developed a valid and reliable self-report questionnaire to measure team cohesion in a work setting. Due to its economic deployment, it is suitable for work contexts with high workload and limited time, especially in health care settings. However, there are still limitations that need to be mentioned. We validated the Erlangen Team Cohesion at Work Scale in a sample of adult nurses in a German University Hospital, but psychometric properties and also factor structure could differ in other samples or settings, since team cohesion is a complex construct with multiple facets. Especially in the light of the COVID-19 pandemic and the exceptionally demanding working conditions resulting from this situation for nurses, team cohesion could play a different role in this context than it does in others. Further research should validate this questionnaire in other work groups and settings. Another limitation is the cross-sectional design. For reliability testing, we were only able to use internal consistencies. Further research should consider longitudinal studies, in order to examine re-test reliability. Since our study was conducted at a German University Hospital, only the German version of our questionnaire could be validated. International studies are needed, to validate the English version.

## Conclusions and practical implications

Team cohesion was found to be a powerful factor for mental health, job performance, job satisfaction and even turnover intention in various settings previously. Therefore, applying valid, reliable and economic measures for team cohesion on a regular basis seems to be of high importance. However, for future research, it is not only of importance to assess and monitor team cohesion but also focus on possibilities for maintaining or increasing team cohesion in the long run. Especially applying team building methods (e.g. team events), communication trainings, trainings on problem management, leadership training or implementing possibilities for social exchange at work (e.g. lunch breaks, reflection rounds [59] etc.) could improve team cohesion. Especially for nurses, where fluctuations are high, special interventions should be explored in the future.

### Electronic supplementary material

Below is the link to the electronic supplementary material.

Supplementary Material 1

Supplementary Material 2

## Data Availability

Data available on request from the authors.

## Abbreviations

COVID-19: Corona Virus Disease 2019
COPSOQ: Copenhagen Psychosocial Questionnaire
TKI: Team Klima Inventar
FITOR: Fragebogen zur individuellen, Team und organisationellen Resilienz
FAT: Fragebogen zur Arbeit im Team
PCS: Perceived Cohesion Scale
ESSI: The ENRICHD Social Support Inventory
ERI: Effort-Reward Imbalance Scale
PHQ: Patient Health Questionnaire
GAD: Generalized Anxiety Disorder
ETC: Erlangen Team Cohesion at Work Scale
CS: Collegial Solidarity
UPM: Unity and Problem Management
M: Mean
SD: Standard deviation
KMO: Kaiser Meyer Olkin
