# Supplementary material for: Development and validation of a questionnaire for measuring team cohesion: the Erlangen Team Cohesion at Work Scale (ETC)
Source: BMC Psychol. 2024 Feb 22;12:91. doi: 10.1186/s40359-024-01583-2 (PMC10885512; doi:10.1186/s40359-024-01583-2)
Supplement: Supplementary file 1 — Supplementary Material 1 [file 40359_2024_1583_MOESM1_ESM.docx]

Additional File 1: Pilot version.

English:

| 1. We support each other |
| --- |
| 1. I do not feel comfortable in this team* |
| 1. We treat each other with respect |
| 1. I feel accepted by my team* |
| 1. We can rely on each other |
| 1. There are members of the team that are being excluded |
| 1. There is a fair distribution of workload within the team |
| 1. Everyone is left to work on their own. |
| 1. There is a good communication within the team |
| 1. We stick together |
| 1. Everyone is free to express their opinion openly |
| 1. We handle problems in a constructive manner |
| 1. There is a sense of “we” among us |
| 1. In case of disagreements, we usually find a good compromise |
| 1. New team members are quickly integrated into the team |

*Dismissed after item analysis

German:

| 1. Wir unterstützen uns gegenseitig |
| --- |
| 1. Ich fühle mich in diesem Team nicht wohl* |
| 1. Wir gehen wertschätzend miteinander um |
| 1. Ich fühle mich von meinem Team akzeptiert* |
| 1. Wir können uns aufeinander verlassen |
| 1. Es gibt Mitglieder im Team, die ausgegrenzt werden |
| 1. Es gibt eine gerechte Verteilung der Arbeitslast im Team |
| 1. Jeder ist bei der Arbeit auf sich alleine gestellt |
| 1. Es gibt eine gute Kommunikation im Team |
| 1. Wir halten zusammen |
| 1. Jeder darf seine Meinung offen sagen |
| 1. Wir gehen konstruktiv mit Problemen um |
| 1. Bei uns herrscht ein „Wir-Gefühl“ |
| 1. Bei Meinungsverschiedenheiten finden wir meistens einen guten Kompromiss |
| 1. Neue Mitarbeiter*innen werden schnell in das Team integriert |

*Entfernt nach Itemanalyse
