# Supplementary material for: Development and validation of a questionnaire for measuring team cohesion: the Erlangen Team Cohesion at Work Scale (ETC)
Source: BMC Psychol. 2024 Feb 22;12:91. doi: 10.1186/s40359-024-01583-2 (PMC10885512; doi:10.1186/s40359-024-01583-2)
Supplement: Supplementary file 2 — Supplementary Material 2 [file 40359_2024_1583_MOESM2_ESM.docx]

Additional file 2: Final Version of the Erlangen Team Cohesion at Work Scale (ETC) in English and German

**Erlangen Team Cohesion at Work Scale, ETC**

**(not validated English Version)**

This questionnaire is about your team cohesion at the workplace. For each of the following statements, please check the answer option that best applies to you and your team.

|  | Strongly disagree  (0) | Disagree  (1) | Neutral  (2) | Agree  (3) | Strongly agree  (4) |
| --- | --- | --- | --- | --- | --- |
| 1. We support each other |  |  |  |  |  |
| 1. We treat each other with respect |  |  |  |  |  |
| 1. We can rely on each other |  |  |  |  |  |
| 1. There are members of the team that are being excluded* |  |  |  |  |  |
| 1. There is a fair distribution of workload within the team |  |  |  |  |  |
| 1. Everyone is left to work on their own* |  |  |  |  |  |
| 1. There is a good communication within the team |  |  |  |  |  |
| 1. We stick together |  |  |  |  |  |
| 1. Everyone is free to express their opinion openly |  |  |  |  |  |
| 1. We handle problems in a constructive manner |  |  |  |  |  |
| 1. There is a sense of “we” among us |  |  |  |  |  |
| 1. In case of disagreements, we usually find a good compromise |  |  |  |  |  |
| 1. New team members are quickly integrated into the team |  |  |  |  |  |

*inverse coded items (0=4, 1=3, 2=2, 3=1, 4=0)

Collegial Solidarity (Item Sum 1,2, 3, 5, 7, 8): 0-24/6 (Number of Items)

Unity and Problem Management (Item Sum 4*,6*,9,10,11,12,13): 0-28/7 (Number of Items)

Total Sum Score Team Cohesion: 0-52/13 (Number of Items)

**Erlanger Fragebogen zur Teamkohäsion am Arbeitsplatz (ETK)**

(validierte deutsche Version)

Bei diesem Fragebogen geht es um ihre Teamkohäsion am Arbeitsplatz. Kreuzen Sie bitte bei jeder Aussage die Antwortmöglichkeit an, die am besten auf Sie und Ihr Team zutrifft.

|  | Trifft überhaupt nicht zu  (0) | Trifft eher nicht zu  (1) | Teils  teils  (2) | Trifft eher zu  (3) | Trifft völlig zu  (4) |
| --- | --- | --- | --- | --- | --- |
| 1. Wir unterstützen uns gegenseitig |  |  |  |  |  |
| 1. Wir gehen wertschätzend miteinander um |  |  |  |  |  |
| 1. Wir können uns aufeinander verlassen |  |  |  |  |  |
| 1. Es gibt Mitglieder im Team, die ausgegrenzt werden* |  |  |  |  |  |
| 1. Es gibt eine gerechte Verteilung der Arbeitslast im Team |  |  |  |  |  |
| 1. Jeder ist bei der Arbeit auf sich alleine gestellt* |  |  |  |  |  |
| 1. Es gibt eine gute Kommunikation im Team |  |  |  |  |  |
| 1. Wir halten zusammen |  |  |  |  |  |
| 1. Jeder darf seine Meinung offen sagen |  |  |  |  |  |
| 1. Wir gehen konstruktiv mit Problemen um |  |  |  |  |  |
| 1. Bei uns herrscht ein „Wir-Gefühl“ |  |  |  |  |  |
| 1. Bei Meinungsverschiedenheiten finden wir meistens einen guten Kompromiss |  |  |  |  |  |
| 1. Neue Mitarbeiter*innen werden schnell in das Team integriert |  |  |  |  |  |

*invers codierte Items (0=4, 1=3, 2=2, 3=1, 4=0)

Kollegiale Solidarität (Summe Items 1,2, 3, 5, 7, 8): 0-24/6 (Anzahl der Items)

Verbundenheit und Problemmanagement (Summe Items 4*,6*,9,10,11,12,13): 0-28/7 (Anzahl der Items)

Gesamtwert Teamkohäsion: 0-52/13 (Anzahl der Items)

©Psychosomatische und Psychotherapeutische Abteilung des Universitätsklinikums Erlangen
